# An efficient control flow validation method using redundant computing capacity of dual-processor architecture

**DOI:** 10.1371/journal.pone.0201127

**Published:** 2018-08-01

**Authors:** Qingran Wang, Wei Guo, Jizeng Wei

**Affiliations:** 1 School of Computer Science and Technology Tianjin University, Tianjin, China; 2 Tianjin Key Laboratory of Advanced Networking (TANK), Tianjin, China; Victoria University, AUSTRALIA

## Abstract

Microprocessors in safety-critical system are extremely vulnerable to hacker attacks and circuit crosstalk, as they can modify binaries and lead programs to run along the wrong control flow paths. It is a significant challenge to design a run-time validation method with few hardware modification. In this paper, an efficient control flow validation method named DCM (Dual-Processor Control Flow Validation Method) is proposed basing on dual-processor architecture. Since a burst of memory-access-intensive instructions could block pipeline and cause lots of waiting clocks, the DCM assigns the idle pipeline cycles of the blocked processor to the other processor to validate control flow at run time. An extra lightweight monitor unit in each processor is needed and a special dual-processor communication protocol is also designed to schedule the redundant computing capacity between two processors to do validation tasks better. To further improve the efficiency, we also design a software-based self-validation algorithm to help reduce validation times. The combination of both hardware method and software method can speed up the validation procedure and protect the control flow paths with different emphasis. The cycle-accurate simulator GEM5 is used to simulate two ARMv7-A processors with out-of-order pipeline. Experiment shows the performance overhead of DCM is less than 22% on average across the SPEC 2006 benchmarks.

## Introduction

In recent decades, microprocessors and embedded devices developed rapidly both in performance improvement and in practical application. At the same time, a growing number of microprocessors are exposed to security threats. Some threats come from hacker attacks which may replace part or all of the binaries. Or they would modify program’s control flow to link to untrusted library functions or jump to malicious code in static or at run time to steal cryptographic keys or private data 11111 Other threats may come from transient faults caused by high-power particle strikes from space environment or circuit crosstalk in electronic circuit. No matter what kind of threats happens, programs could not perform their function correctly. The key point of trust computing is ensuring programs can be executed along the designed control flow paths at run time and do exactly what we want them to do.

Current control flow validation methods can be mainly classified into two kinds. One kind is designing extra hardware devices in chip to monitor, compute and validate run-time control flow information [1–4]. General thought of this method is analyzing the source code of application programs in static to get control flow information and then generate signatures using the more detailed physical addresses extracted from corresponding binaries [3, 4]. The pre-generated signatures are usually stored in a special designed memory. When programs run, the extra hardware device would regenerate signatures to compare with the pre-generated signatures to find mismatches. If any mismatches appear, the hardware device will stop the system at once and report wrong messages. This method has high fault coverage rate, but would introduce high performance overhead because it checks every potential control flow edges. Besides, the extra hardware device also occupies a large chip area. Average performance penalty of this kind of control flow validation method is approximately 75% according to the statistics of Khudia et al [2]. So it is of great urgency for this method to reduce checking times. Some traditional validation methods such as REV (Run-time Execution Validator) [1], REM (Run-time Execution Monitoring) [3] and some other designs with extra hardware [4] belong to this kind. The other way to authenticate control flow paths is using a pure software-based technique which can reduce the extra needed chip area introduced by hardware devices and speed up the validation procedure to some degree. Method CFCSS (Control Flow Checking by Software Signatures) [5] belongs to this kind. This kind of method does not need any extra hardware support but most of them need to modify assembly source code or binary files to insert self-validation code. At the same time, this method also needs to design a self-validation algorithm to help verify the correctness of the control flow. Pure software-based validation technique introduces less performance overhead but has lower fault coverage rate because software cannot detect attacks which are directed against hardware faults. And the inserted validation code itself is exposed to attacks as well. So it is hard to design an efficient control flow protection algorithm basing on traditional architecture.

In dual-processor or multi-processor architecture, processors are not always working in parallel. Sometimes when one processor is executing calculation-intensive instructions, the other processor is executing memory-access-intensive instructions. Running lots of memory-access-intensive instructions in one processor may cause serious cache misses and block the pipeline. In this condition, the blocked pipeline resource can be used to do control flow validation tasks by the other processor. This method does not need a special hardware to do computing or comparing work and can take full use of the redundant computing capacity in the dual-processor architecture.

In this paper, we propose a completely new control flow validation method named DCM using the potential redundant computing capacity of dual-processor architecture to do control flow validation task. The hardware part of DCM is composed of several registers and controlling circuit without introducing any extra hardware devices to do computing tasks. We design a scheduling module in each processor to monitor the other processor’s status and prepare to schedule the validation tasks between two processors. The control flow information of every validation tasks is designed in a unified format. In this way the validation task can be precessed smoothly by each processor without any obstacle. To further improve the efficiency and reduce the validation times, we also design a software-based self-validation algorithm. The designed self-validation algorithm fulfills its function by adding extra code to assembly source code. The added code does not validate every control flow edge, but only validates control flow in loops. Because if we let DCM validate every control flow edge, one control flow path in a loop would be validated several times before the program jumps out of this loop. It is a waste for system to spend so much computing resource on the same validation task. The software part of DCM can do the same validation task just as the hardware part do. And the using of software method can prevent the waste of time that hardware method costs when scheduling the computing resource between two validation tasks. Therefore the combination of both hardware method and software method is an efficient way to speed up validation procedure and protect control flow with different emphasis.

The remainder of this paper is organized as follows. In Section 2, we introduce some classic methods of control flow authentication and the possibility of utilizing the redundant computing capacity. Then the detailed designs and the implementation of DCM architecture are described in Section 3, including the designs of some key components and a special dual-processor communication protocol. In Section 4, we will talk about the special self-validation algorithm in detail. And Section 5 gives the experiment and evaluation results of the performance overhead of DCM. In the end we will conclude this paper in Section 6.

## Related works

### Control flow authentication

CFA (Control Flow Authentication) method generally relies on two ways to authenticate CFG (Control Flow Graph). One way is checking basic block integrity and the other way is validating control flow path [1]. The most famous TPM (Trusted Platform Module) [6] belongs to the first one. TPM encrypts its data and checks the binaries’ hash integrity before programs run. Some other CFA methods also need to validate basic block integrity, but differ from each other in implementation [1, 3, 4, 7–9].

Checking program’s integrity prior to its running cannot detect attacks or faults appearing at run time. To overcome this limitation, some CFA methods tried to insert two or more checkpoints in programs and validate control flow edges at these particular checkpoints [10, 11]. But if attacks or faults appear between two checkpoints, this method fails to detect them as well.

The most widely used CFA pattern is validating control flow at run time. Classic methods extract control flow paths or signatures of basic blocks in static and write them into binaries or store in a special memory. When one program is being executed, an extra hardware component validates control flow paths to ensure the program run correctly [12, 13]. One typical method is SIS (Signatured Instruction Streams). In SIS, the application program is appropriated partitioned into several sub-programs at compile time. And some verification information such as signatures are also inserted into these smaller sub-programs. When program runs, a special built-in hardware regenerates these verification information and finds the mismatches [14]. This classic CFA method has high fault coverage rate but needs extra hardware support and also needs to modify binary files. What’s more, the average performance overhead of this CFA method can be more than 75% [2]. To accelerate checking procedure, Aktas E et al adds a special purpose cache in processor with out-of-order pipeline in REV [1]. This special cache can indeed speed up the validation procedure but it also introduces extra hardware overhead.

To avoid extra hardware overhead, CFCSS [5], ACS [2] and CFI (Control-Flow Integrity) [15] employ a pure software method to validate control flow paths. CFCSS assigns every basic block a unique signature value s_*i*_ and a unique signature difference value d_*i*_. These two values can be used to compute a particular validation value to help validate control flow paths. ACS divides a program into pieces of regions, and only validates control flow edges at the end of a region. ACS can reduce validation times efficiently compared to those CFA methods which need extra hardware support. However, if any attack or fault appears inside a region, ACS cannot protect system anymore. Because damaged data has already been written into memory.

### Utilization of the redundant computing capacity

Utilizing the redundant computing capacity is a widely adopted method of exploring potential computing capacity of system or processor unit [16]. Similar techniques have already been researched in GPGPU (General Purpose GPU) domain [10, 11]. Jeon et al analyzed the possibility of utilizing the redundant computing capacity of GPGPU in Warped-DMR (Warped Dual Modular Redundancy) [11]. Because of the trend of using GPGPU to do general computing tasks, some threads of GPU which are originally designed to process large data load in parallel are idle. Warped-DMR uses this underutilized computing capacity to detect faults appearing at run time.

Generally, there are two patterns in which two processors can work together. First one is a kind of master-slave pattern. In this pattern, one processor (master) does the general computing task and the other processor (slave) monitors the status of system. This kind of master-slave architecture is widely adopted in embedded system especially in automotive electronics domain to ensure system security [17]. In the second pattern, two same processors are used to do the same computing task. But the computing results of each processor are compared to find difference. More commonly, two same processors usually execute different computing tasks to speed up computing procedure. In this paper, we mainly discuss how to utilize the redundant computing capacity of two same processors which execute different computing tasks with out-of-order pipeline.

In out-of-order pipeline, after being fetched in order, instructions are dispatched to wait in reservation stations. One instruction has to wait in reservation station until its all related operands are available and then processor would execute this instruction. Although all instructions are fetched in order, they are executed in out-of-order manner. The purpose of this dispatching manner is ensuring every instruction has all necessary operands prior to executing. In this way can reduce stalls in pipeline. Because the related operands of one instruction may come from another instructions, inserting stalls into pipeline to wait for data-related operands is hard to avoid. So out-of-order processor usually has to fetch a bunch of instructions at one time and inserts some non-data-related instructions into blocked pipeline trying to take full use of the pipeline resource. But processor cannot always take full use of all the blocked cycles in pipeline. When fetching a bunch of memory-access-intensive instructions, processor cannot find enough non-data-related instructions to insert into pipeline in one fetching cycle. It certainly will cause a lot of stalls in pipeline and the system would need a long time to overcome it.

The situation discussed above is hard to overcome for the system with only one processor. In dual-processor architecture, when one processor is blocked because of many memory-access-intensive instructions, it is a chance for the other processor to utilize the redundant computing capacity to do control flow validation tasks.

### Explore the possibility of utilizing the idle computing cycles

Before doing more work, an experiment was designed to explore how to make better use of the idle computing cycles. Based on the previous analysis, the blocked situation is most likely to occur when one processor runs a large number of memory intensive instructions. Nine test programs was selected form SPEC 2006 benchmark and was sorted in oder of memory access rate. The experiment was did on gem5 simulator platform and the only one ARMv7-A processor was simulated in system call mode. More experiment details can be referred in ‘Performance Evaluation’ section.

As S11 Fig shows, the degree of pipeline blocked cycles changes along with the degree of direct memory access changing. And in general, this trend of changing presents a linear relationship. Although in some test programs, this trend presents a trend of non increasing. We think this situation may be caused of caches. The three test-benches with most obvious downward trend are ‘462-libquantum’, ‘456-hmmer’ and ‘429-mcf’. And these three test-benches contain 10, 14 and 11 complete loops respectively, which is more than the average loops number of the benchmark SPEC2006. And there may be more memory access instructions are placed in loops than other test-benches. So the memory addresses those instructions trying to access are more fixed. The presence of caches can help reduce the times of fetching data from memory. In this situation, the blocked cycles could be less than other test-benches. In addition to this special situation, the trend of changing in S11 Fig presents a linear relationship in general. So it is possible that the higher degree of one program’s memory access instructions that one processor running, the higher possibility that this processor has more blocked computing cycles that can be utilized. At the same time, we also found that in two cases, the idle cycles of one processor can be fully utilized. The first case is when one processor executes a lot of memory access instructions, we can utilize the blocked computing cycles. And the second case is when one processor’s task queue is empty and this processor is in idle mode. This situation is not very common, but from the results of the experiment, the idle time rate of one processor can reach 0.06% on average. And this is also a valuable resource that can be well utilized.

## Designs and implementation of hardware part of DCM

In this section, we will introduce the architecture of hardware part of DCM and the most important component—Scheduler in DCM. Then the manner in which two processors communicate with each other will be discussed. In the end of this section, we will talk about how DCM divides control flow validation tasks and assigns them to each processor.

### Overview of DCM hardware architecture

In hardware part of DCM, there are two same out-of-order processors. Each processor has a logical module—Scheduler which is used to monitor two processors’ status and assign control flow validation tasks to each processor. Two Schedulers in each processor are all the same and we will only discuss the function of the Scheduler in the view of CPU1 in the rest of this paper.

S1 Fig shows the overall architecture of DCM. The two block diagrams above mainly indicate the architecture of the hardware part of DCM. Each processor has a special added component named Scheduler. The Scheduler is the main function unit used to schedule computing resource to do validation task. The Scheduler is composed of controlling circuit and two register stack which are entry stack and history stack to help store control flow entries temporarily. And the CFG memory is designed to store control flow entries for a long time (time limit for program running). When program is being executed on system implementing DCM, the Scheduler in CPU1 will wait to receive control flow validation tasks from CPU1 itself. If CPU1 has one control flow edge waiting to be validated, it will send the source address and the target address of this control flow edge to its Scheduler. Then the Scheduler packs these addresses into an entry to store in its own entry stack temporarily. After that, the Scheduler will monitor the status of two processors to find chances to validate this control flow entry.

The key point of validating control flow edge is to ensure that programs run along the correct control flow path as designed. The following procedure of S1 Fig describes the approximately procedure of constructing a self-validation binary which is needed in software part of DCM. We designed a special pre-processing tool to analyze and extract correct control flow entries from binaries and stored these entries into CFG memory as golden rules to validate run-time generated control flow entries. When validating a control flow entry, processor compares this entry with the entries stored in CFG memory to check if any illegal branch appears.

### The scheduler in DCM

In DCM, the Scheduler plays an extremely important role. It stores control flow entries which are waiting to be validated in entry stack and stores entries which have been successfully authenticated in history stack to perform the role of a cache to some degree. Besides, the Scheduler in one processor also needs to communicate with the other processor’s Scheduler. So it is necessary to figure out the architecture of the Scheduler.

The Scheduler is mainly composed of entry stack, history stack and a controller module as S2 Fig shows. Entry stack is made up of several registers which are organized in FIFO (first in first out) manner. It also has a shadow stack named history stack storing entries which are authenticated successfully recently. Before processor sends a new entry to entry stack, the Scheduler compares this new entry with entries which are stored in history stack to check if this control flow entry has already been authenticated successfully before. If the new entry has already been authenticated successfully before, there is no need to validate it twice. Controller module is used to assign validation tasks to the other processor. It writes entries to a particular area of CFG memory and sends processing signal to the other processor when that processor has redundant computing capacity. It also sends idle signal to the other processor’s Scheduler to inform that this processor has redundant computing capacity and receives idle signal from the other processor vice versa. Controller module also communicates with program counter (PC). When processor needs to validate a control flow entry, it saves the current PC value and loads PC with addresses of instructions which are used to validate entries. When a validation task is completed, it writes the original PC value back to PC and lets processor continue to execute the rest instructions.

### DCM communication protocol

In dual-processor architecture, the communication between two processors is very important. In DCM, we designed a special communication protocol to guide two processors to exchange information with each other. Since the protocol is quite complex, we will describe the status transformation procedure using a FSM (Finite State Machine) *M*_*DCM*_ which is shown in Table 1. And the corresponding symbol definition can be seen in Table 2.

**Table 1:** FSM of DCM communication protocol.

*FSMM*_*DCM*_ = (*S*, ∑, *δ*, *s*_0_)
*S* = {*s*_0_, *s*_1_, *s*_2_, *s*_3_, *s*_4_, *s*_5_}
$\sum =\{a,b,\bar{b},c,d,e_{1},e_{2}\}$
1. *δ*(*s*_0_, Λ) = *s*_1_
2. *δ*(*s*_1_, *a*) = *s*_2_
3. $\delta \left(s_{2},\bar{b}\right)=s_{4}$
4. $\delta \left(s_{2},\neg \bar{b}\right)=s_{3}$
5. *δ*(*s*_2_, *d*) = *s*_5_
6. *δ*(*s*_3_, *c*) = *s*_4_
7. *δ*(*s*_3_, *e*&¬*b*) = *s*_5_
8. *δ*(*s*_4_, *b*) = *s*_1_
9. *δ*(*s*_4_, ¬*b*) = *s*_3_
10. *δ*(*s*_5_, *b*) = *s*_1_
11. *δ*(*s*_5_, ¬*b*) = *s*_2_

**Table 2:** Definition of symbols in *M*_*DCM*_.

Status Name	Representation
*s*_0_	System start
*s*_1_	CPU1 idle status
*s*_2_	Entry handling status
*s*_3_	Waiting status
*s*_4_	CPU1 computing status
*s*_5_	CPU2 computing status
*b*	Local stack is empty
$\bar{b}$	Local stack is full
*c*	An entry waits overtime
*d*	CPU2’s activity queue is empty
*e*	CPU2 triggers computing chance

In *M*_*DCM*_, the S is a set of system statuses which consists of 6 different statuses that system would switch to. ∑ is a set of finite alphabet and each item in set ∑ represents a possible system condition that can affect system status. The *δ* of *M*_*DCM*_ indicates a function which switches between two statuses. And the function format *δ*(*s*_*i*_, *a*) = *s*_*j*_ indicates when system is in status *s*_*i*_ and meets the condition a, then system will switch to status *s*_*j*_.

In the perspective of CPU1, when system starts up, *M*_*DCM*_ switches to ‘CPU1 idle status’—*s*_1_ (‘idle’ here doesn’t mean CPU is idle but mean CPU does not have any validation tasks). If CPU1 needs to validate a control flow edge, it is impossible for CPU1 to stall all the rest instructions to do validation task at once. On the contrary, CPU1 packs this control flow edge’s source address and target address in an entry, and sends this entry to its Scheduler to handle. Then *M*_*DCM*_ enters ‘entry handling status’—*s*_2_. At status *s*_2_, CPU1 continues executing its rest instructions and its Scheduler checks whether its entry stack is full. If entry stack is full, CPU1 has to validate the first entered entry in stack at once. But if the Scheduler finds CPU2’s activity queue is empty, CPU1’s Scheduler will send its control flow entries to CPU2 to validate as well. Entry stack consists of a few registers used to store entries waiting to be validated temporarily and set a threshold value of waiting time. If any entries wait in entry stack overtime, local processor has to validate this control flow entry at once. No matter when CPU1 does validation tasks, *M*_*DCM*_ switches to CPU1 computing status *s*_4_. Once one validation task is completed, the Scheduler checks whether the entry stack is empty. If stack is empty, *M*_*DCM*_ switches to status *s*_1_ to wait for a new entry. Otherwise, *M*_*DCM*_ switches to waiting status *s*_3_. When system enters waiting status, it means there is not enough redundant computing capacity for system to do validation tasks and system has to wait for next validation chance.

But what is the proper time to do control flow validation? As discussed above, only in two situations can we use the system’s redundant computing capacity to do validation task. In the first situation, when CPU1’s Scheduler finds CPU2 is in idle status, it stores one control flow entry in a particular area of CFG memory and sends a processing signal to CPU2. Then CPU2 loads this control flow entry to its entry stack and then does validation task by executing divided instructions generated by the Scheduler in CPU2. In the second situation, when one processor’s Scheduler finds its processor pipeline has blocked cycles, it sends trigger signal to the other processor just as procedure *δ*(*s*_2_, *d*) = *s*_5_ and procedure *δ*(*s*_3_, *e*&¬5) = *s*_5_ indicates.

### Validation task division

One control flow validation task cannot be completed by simply executing a single instruction. When validating a control flow entry, processor should search the CFG memory for the matched entry to conform the correctness of this entry. In this procedure, processor may execute lots of load instructions and lots of compare instructions. To achieve the validation task, DCM divides these instructions into several pairs. Each pairs consists of one load instruction and one compare instruction. When validating entries, processor receives the divided pairs from the Scheduler and executes them separately. It is good that a single validation task is completed in one turn. But if one processor does not finish a validation task in one turn and the original blocked instructions are ready to be executed, adding stalls in pipeline till all the pairs are executed is not a good choice. DCM solves this problem by executing every instruction pairs in atomic manner. DCM finishes the execution of the latest instruction pair and lets pipeline go on executing its original rest instructions. If the result of the latest instruction pair indicates this control flow is legal, we do not need to execute the rest pairs. But if we cannot prove the legality of this control flow edge, the rest instruction pairs should still be executed in the next validation turn.

## Software-based self-validation algorithm

To reduce the validation times that DCM performs, we designed a software-based self-validation algorithm to do less-critical validation tasks by program itself. And this self-validation algorithm is DCM’s software part. In this Section, we will explain the principle of the self-validation algorithm in detail and then describe the procedure of constructing a special self-validation binary.

### Principle of self-validation algorithm

There are two main reasons why we use the special self-validation algorithm to protect control flow in loops. First, one control flow entry in a loop would be validated several times before the program jumps out of this loop. The repeated validation tasks waste precious computing resources and may introduce high performance overhead. Second, if DCM’s history stack is big enough, it is possible for DCM to use the history stack as a ‘cache’ which can help DCM skip the repeated control flow entries. But the number of control flow entries in a loop is different in different programs, we also cannot have enough registers to form an extremely large stack. So it is necessary to find another method to protect control flow in loops.

The self-validation algorithm helps system protect control flow in loops by inserting self-validation code to assembly source code to check validation values before every BBs (basic block). We improve the CFCSS’s self-validation algorithm to meet the need of protecting loops. Three validation values are assigned to each BB which are *W*, *d* and *D* for BB weight, weight difference and BB distinction, respectively. The signature in CFCSS is not used because control flow correction is our main consideration and basic block integrity validation costs far more resources than only validating control flow. The main idea of our self-validation algorithm is using *W* and *D* of current BB with *d* of target BB to compute a temporary BB weight *W*_*t*_ before program jumps to target BB. If *W*_*t*_ equals to target BB’s *W*, we think this control flow edge is legal.

Every directly linked path form one BB to another BB has a path weight which is 1. BB weight *W* is the max value adding all path weights along the longest path from start BB to this BB. Other two validation values assigned to each BB helping to compute BB weight are *d* and *D*. The *d* represents difference between control flow source BB and target BB. The *D* represents distinction between two BBs which jump to one same BB. When a control flow path flows from BB *V*_*i*_ to BB *V*_*j*_, *V*_*j*_’s *d*_*j*_ equals to *W*_*i*_ ⊕ *W*_*j*_, and *V*_*j*_’s *D*_*J*_ equals to *W*_*j*_ ⊕ *W*_*j*_. In a special situation when *V*_*j*_ has more than one precursor BB which are BB *V*_*i*1_ and BB *V*_*i*2_, *V*_*j*_ first chooses one standard precursor BB *V*_*i*1_ and then computes *d*_*j*_ as *W*_*i*1_ ⊕ *W*_*j*_. At the same time, *V*_*i*1_ and *V*_*i*2_ must modify their *D* values according to *V*_*j*_’s standard precursor BB *V*_*i*1_. *V*_*i*1_’s *D*_*i*1_ equals to *W*_*i*1_ ⊕ *W*_*i*1_ and *V*_*i*2_’s *D*_*i*2_ equals to *W*_*i*1_ ⊕ *W*_*i*2_. In this way, we can compute all the three kinds of validation values of each BB and specify different control flow paths in a formal manner.

We assume if a control flow from *V*_*i*_ to *V*_*j*_ is legal, the computed weight value *W*_*i*_ ⊕ *d*_*j*_ ⊕ *D*_*i*_ always equals to *V*_*j*_’s weight *W*_*j*_. In S3 Fig, when a control flow path flows from *V*_3_ to *V*_4_, computed weight value is *W*_3_ ⊕ *d*_4_ ⊕ *D*_3_ which is *W*_3_ ⊕ *W*_2_ ⊕ *W*_4_ ⊕ *W*_2_ ⊕ *W*_3_ which equals to *W*_4_. So this control flow is legal. When control flow flows from *V*_1_ to *V*_4_, computed weight is *W*_1_ ⊕ *d*_4_ ⊕ *D*_1_ = *W*_1_ ⊕ *W*_2_ ⊕ *W*_4_ ⊕ *W*_1_ ⊕ *W*_1_ which doesn’t equal to *W*_4_. In this situation, we think this control flow is illegal.

### Procedure of constructing a self-validation binary

S4 Fig shows the procedure of constructing a special binary implementing the self-validation algorithm. First, we divide program’s assembly source code into BBs by finding instructions which terminate the BB (such as branch, jump, return, exit instruction etc.). Then we construct a directed graph describing the relationship among BBs. Because every BB in a loop is strongly connected in directed graph, we just need to find out every strongly connected sub-graph and these strongly connected graphs are the loops we need. Then we insert self-validation instructions without validation values into source code to occupy position of further validation code (if we do not insert these empty instructions, physical control flow addresses are incorrect after disassembly). Second, we compile the source code and get first version of binary. After that, we disassemble this binary to get the physical addresses of each control flow entry and compute validation values. Then we add validation values to self-validation instructions to replace empty instructions which are inserted prior to disassembly. In the end, we compile source code and get the final binary to run.

The self-validation instructions are shown in S5 Fig. In a loop, we add designed assembly instructions on the top of every basic block. Two registers *r*_9_ and *r*_10_ store latest validated values *W* and *D* respectively. When the value of *W* is computed, this value should be compared with control flow target BB’s weight value *W*_*j*_. If any mismatches appear, system stops all the tasks at once and jumps to error handling module. In the end, new value of *D* is updated using the *W*_*j*_ of current validated BB *V*_*j*_.

## Performance evaluation

The performance overhead of proposed control flow validation method with DCM was evaluated through simulation using the cycle-accurate simulator GEM5 [18] in SE (system call) mode modeling an ARMv7-A profile of ARM architecture with benchmark SPEC2006. The processors we used is two out-of-order processors with two levels of caches. And this simulator was modified extensively to simulate DCM’s function. The SimPoint [19] technique is also used to accelerate simulation speed. The more detailed simulation configurations can be found in Table 3. Simulating DCM on the ARM platform, some additional hardware resources need to be introduced. Some main additional hardware resources are introduced in Table 4. Both entry stack and history stack are designed with registers. The depth of each stack is 5 and the entry stack’s waiting time threshold is 6.x CPI time which is compared with normal system. The memory space overhead of the specially designed CGF_Memory is less than 5KB. And each control flow entry in CFG_Memory requires the storage space of 16B to store information. In some main stream ARM processors, such as Cortex-A7, the size of L1 and L2 cache is between 8KB to 64KB. The additional hardware resources in DCM is even far more less than the cache size. Even the largest CFG_Memory unit only needs less than 5KB space. So the additional hardware resources the DCM needs are very small.

**Table 3:** Simulation configuration.

Architecture Parameter	Configuration
Simulation Type	System Call mode
Pipeline	Out Of Order
CPU Clock	1GHz
Cache	32KB L1 DCache
	32KB L1 ICache
	128KB L2 Cache
Memory size	7GB

**Table 4:** Additional hardware resources.

Hardware Resource	Size	Function
Entry Stack	20B	Store entries temporarily
History Stack	20B	Used as a cache
CFG Memory	5KB	Store correct CFG entries
One formal control flow entry	16B	The unified format entry

We expect that the performance loss of DCM would be small due to two reasons. First, implementing DCM on system to validate every control flow edge during the execution of program would introduce high performance overhead. Based on this view, we design a special software-based self-validation algorithm to help reduce validation times by protecting loop structures by program itself. Second, in dual-processor architecture, if one processor needs extra computing capacity to do control flow validation tasks and the other processor’s pipeline happens to have lots of blocked cycles, it would improve DCM’s performance by reducing rescheduling time. It means we can let two processors run two different kinds of programs separately. One processor executes memory-access-intensive program, while the other processor executes calculation-intensive program. In this way, the other processor would get more chances to use the blocked pipeline cycles to do validation tasks.

For evaluation, we use the IPC(Instructions per Cycle) to measure performance overhead. In S6 Fig, we simulate two kinds of systems implementing DCM running a subset of SPEC2006 and compare the IPC of them with the IPC in regular system without DCM. One pattern runs benchmark with self-validation algorithm support and the other pattern does not use self-validation algorithm. Results show average performance overhead of DCM system without self-validation algorithm is close to 40% while the performance overhead of DCM system with self-validation algorithm is less than 22% on average. The decrease of performance overhead is mainly due to the fact that the software-based self-validation algorithm helps protect the control flow edges in loops which are usually validated several times before program jumps out of the loop.

Every benchmark of SPEC2006 are also analyzed. Three calculation-intensive benchmarks and three memory-access-intensive benchmarks are selected in order to find best combinations that would reduce the performance overhead of DCM. In S7 Fig, we combine two memory-access-intensive benchmarks and compute each processor’s performance overhead compared to running two same benchmarks on two same processors with self-validation technique support. It can be seen that in some combinations the performance overhead of two processors increases together and in other combinations the performance overhead of two processors decreases together. In the combination of *mcf* and *sleng*, one processor’s performance overhead increases and the other processor’s performance overhead decreases. There is not a uniform trend of performance changing when combining two memory-access-intensive benchmarks. Same situation happens when we combine two calculation-intensive benchmarks in S8 Fig. The reason for the ambiguous results of combining two benchmarks of the same type might be that the redundant computing capacity is similar between two processors executing benchmarks with the same type. In the case of combining two calculation-intensive benchmarks, there might be not enough idle cycles for processors to schedule when one processor needs to do validation task. In the case of combing two memory-access-intensive benchmarks, there might be too much scheduling penalty when pipelines of two processors are both blocked.

But things are different when we combine one calculation-intensive benchmark with one memory-access-intensive benchmark. In S9 Fig, it can be seen that the performance overhead of every calculation-intensive benchmark decreases compared to only running the benchmark by itself and the performance overhead of every memory-access-intensive benchmark increases at the same time. This is because the processor running memory-access-intensive benchmark does much more validation tasks from the other processor and the processor running calculation-intensive benchmark does fewer validation tasks compared to only running the benchmark by itself.

We also analyze the source code’s length overhead caused by inserting the self-validation code. Because we only insert protecting code upon BBs in loops, we find that there are only fewer than 10 BBs need to be inserted self-validation code in more than one thousand BBs in our experiment. In S10 Fig, we can see the average code length overhead is less than 1% compare to the 35% overhead of the self-validation algorithm used in ACS [2].

## Conclusions

In this paper, we proposed a new control flow validation method named DCM using the redundant computing capacity of dual-processor architecture to do control flow validation tasks. We also designed a special dual-processor communication protocol and assigned a ‘Scheduler’ component to each processor to monitor status of two processors and assign validation tasks to two processors. To further improve the efficiency, we also proposed a software-based self-validation algorithm to help reduce validation times by inserting self-validation code to do some validations by program itself and this algorithm can decrease DCM’s performance overhead by 17.69%.

To evaluate DCM’s performance overhead, we implemented DCM on GEM5 simulator with two out-of-order processors and found performance overhead is less than 22% with software-based self-validation algorithm support on average.

In this work, we used the dual-processor architecture to explore the possibility of utilizing the redundant computing capacity of two absolutely independent processors. We want to create a relatively stable environment to research the parameters changes when running programs on a single processor in a dual-processor architecture. But in dual-core processor architecture, two cores can share some common resource and this architecture can simplify the communication protocol and reduce the hardware cost. In the following research, exploring the possibility of implementing DCM on two-core processors or on hyper-threaded cpus is a good direction.

## Supporting information

S1 Fig Overview of DCM architecture. (TIF)

S2 Fig Overview of DCM architecture. (TIF)

S3 Fig Principle of detecting illegal target. (TIF)

S4 Fig Procedure of constructing a self-validation binary. (TIF)

S5 Fig Self-validation code. (TIF)

S6 Fig Performance overhead of DCM. (TIF)

S7 Fig Combination of two memory-access-intensive benchmark. (TIF)

S8 Fig Combination of two calculation-intensive benchmark. (TIF)

S9 Fig Combination of memory-access-intensive benchmark with calculation-intensive benchmark. (TIF)

S10 Fig Increase of source code length caused by inserting self-validation code. (TIF)

S11 Fig Changing trend of processor’s blocked cycles. (TIF)

S1 File Output statistic data of GEM5 while running different benchmarks. (ZIP)

S2 File Part of modifying code of GEM5. (ZIP)
