# Supplementary figures and images for: An efficient control flow validation method using redundant computing capacity of dual-processor architecture
Source: PLoS One. 2018 Aug 1;13(8):e0201127. doi: 10.1371/journal.pone.0201127 (PMC6070227; doi:10.1371/journal.pone.0201127)

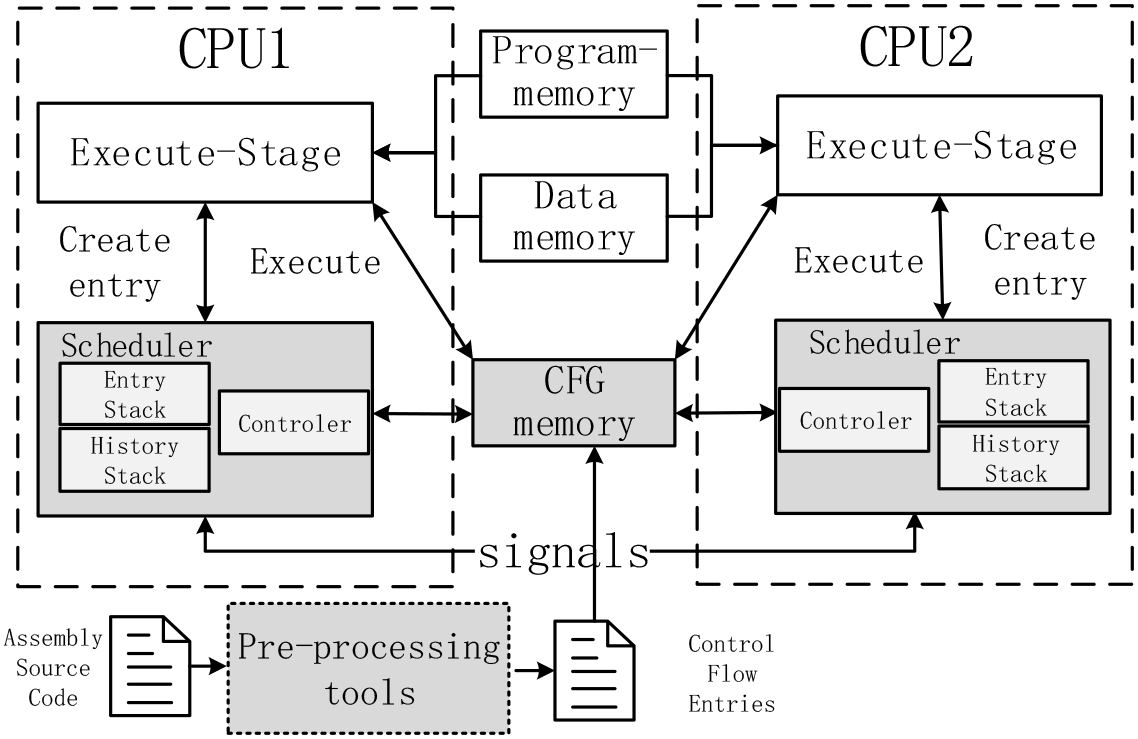

Supplement: S1 Fig — (TIF) [file pone.0201127.s001.tif]

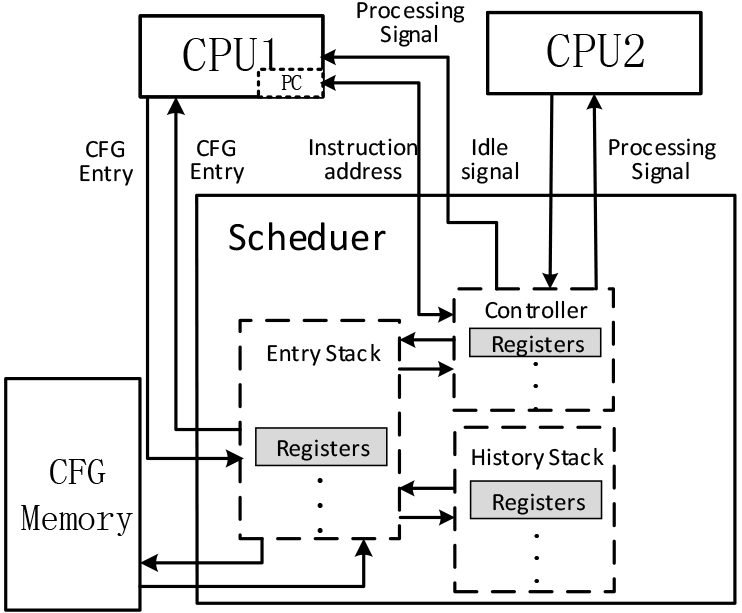

Supplement: S2 Fig — (TIF) [file pone.0201127.s002.tif]

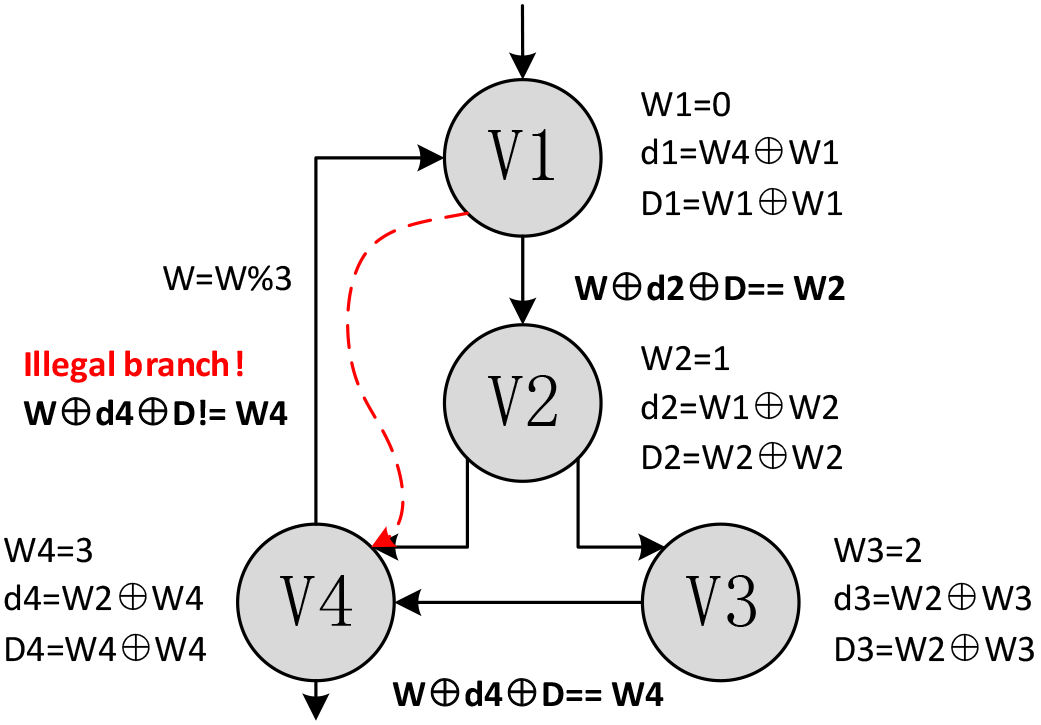

Supplement: S3 Fig — (TIF) [file pone.0201127.s003.tif]

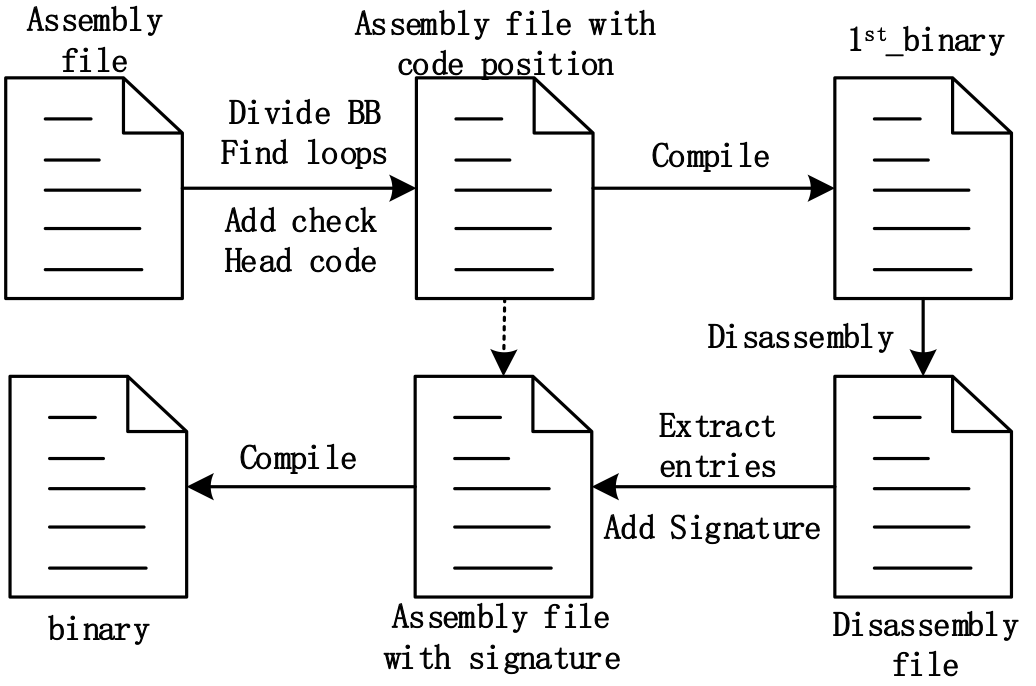

Supplement: S4 Fig — (TIF) [file pone.0201127.s004.tif]

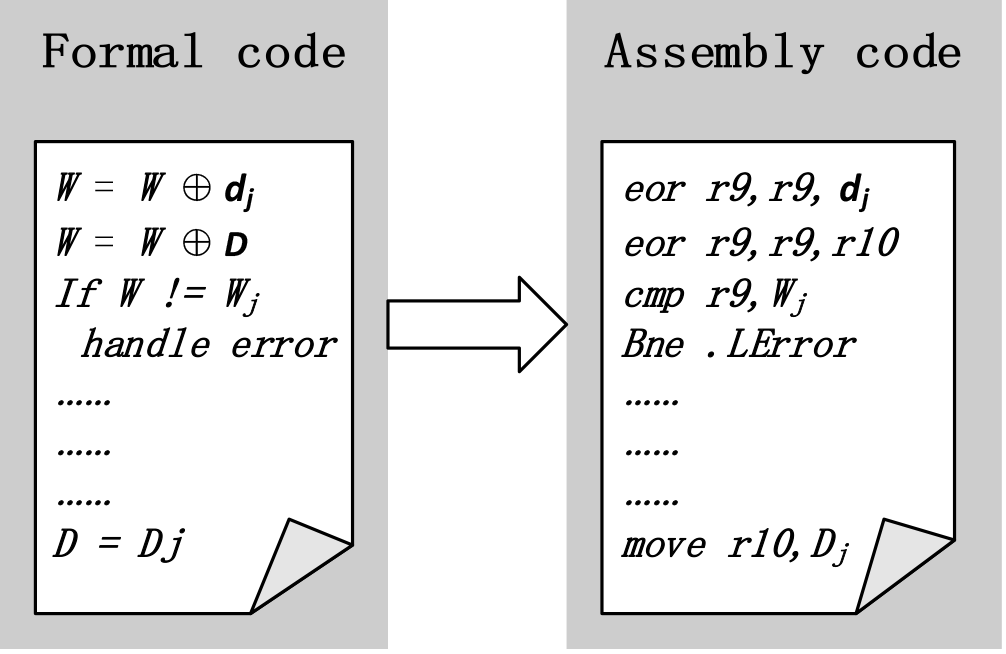

Supplement: S5 Fig — (TIF) [file pone.0201127.s005.tif]

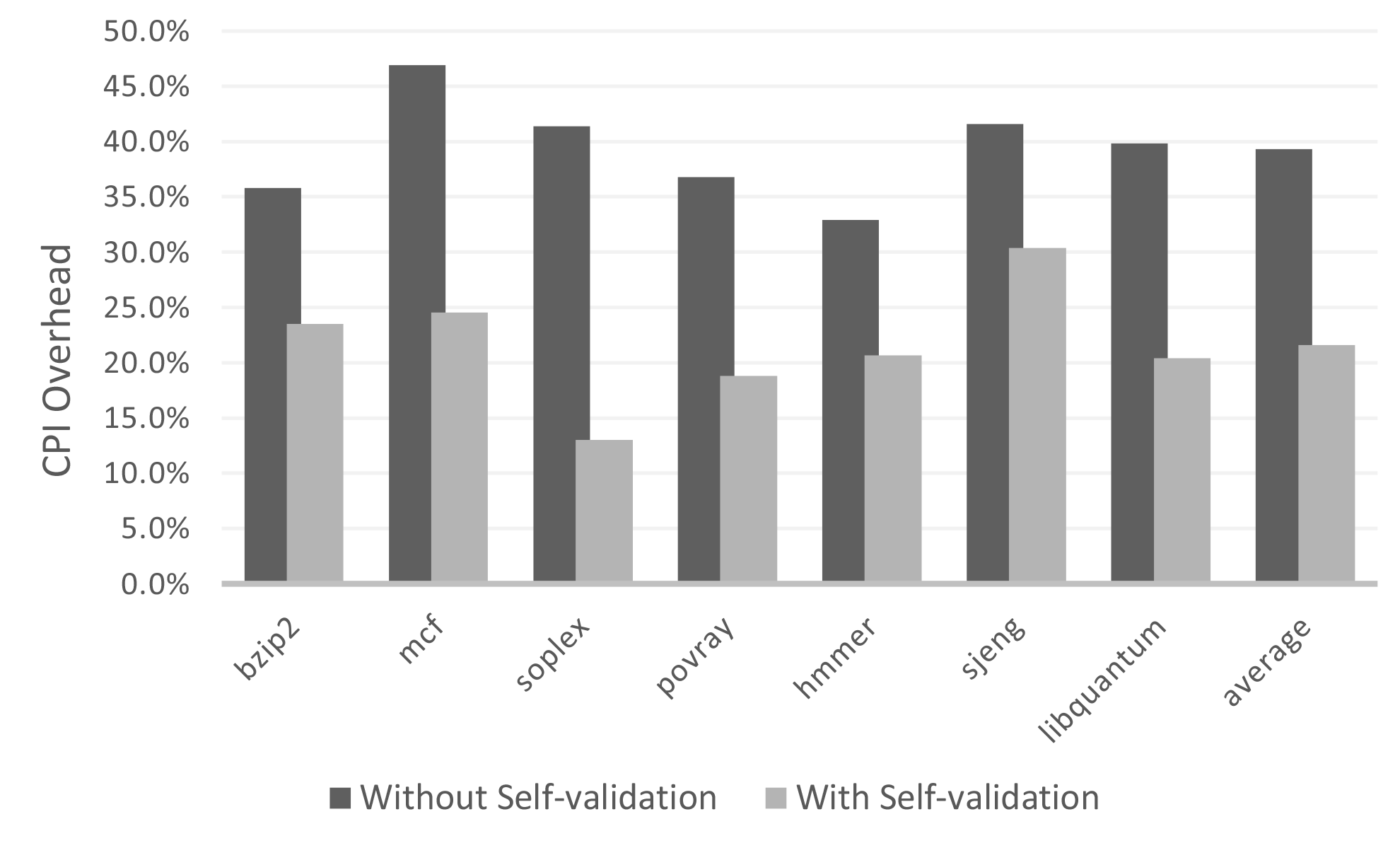

Supplement: S6 Fig — (TIF) [file pone.0201127.s006.tif]

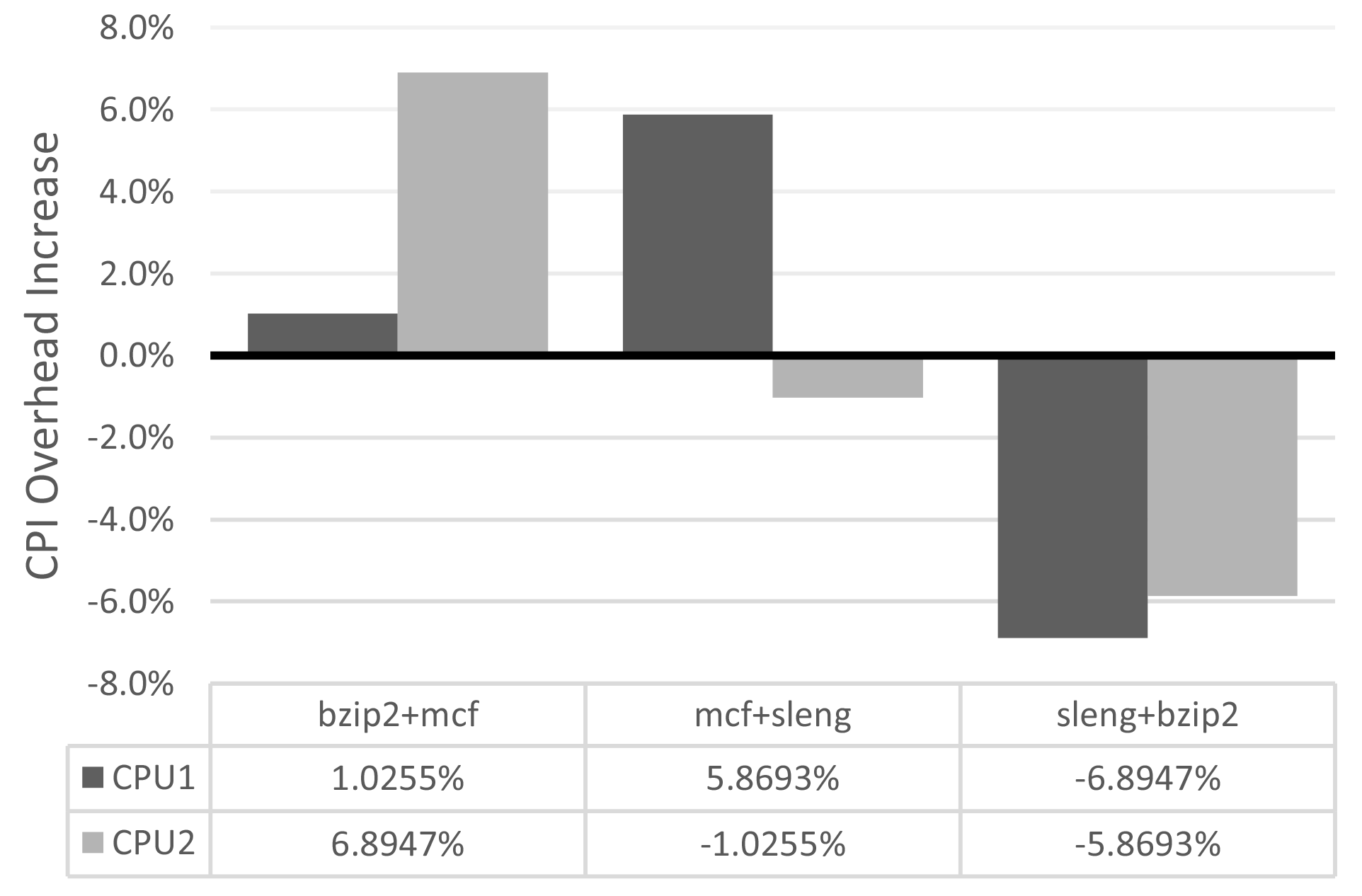

Supplement: S7 Fig — (TIF) [file pone.0201127.s007.tif]

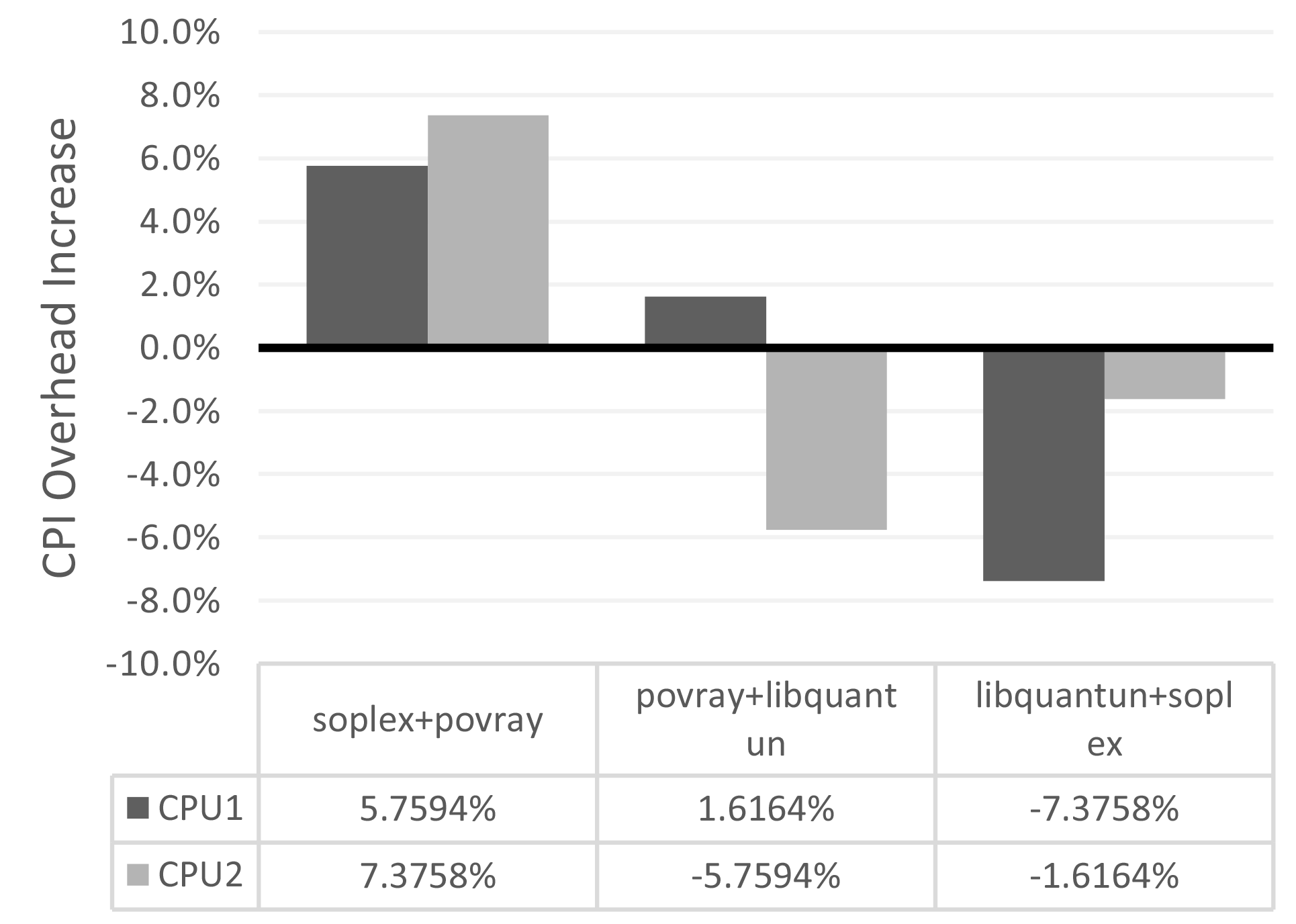

Supplement: S8 Fig — (TIF) [file pone.0201127.s008.tif]

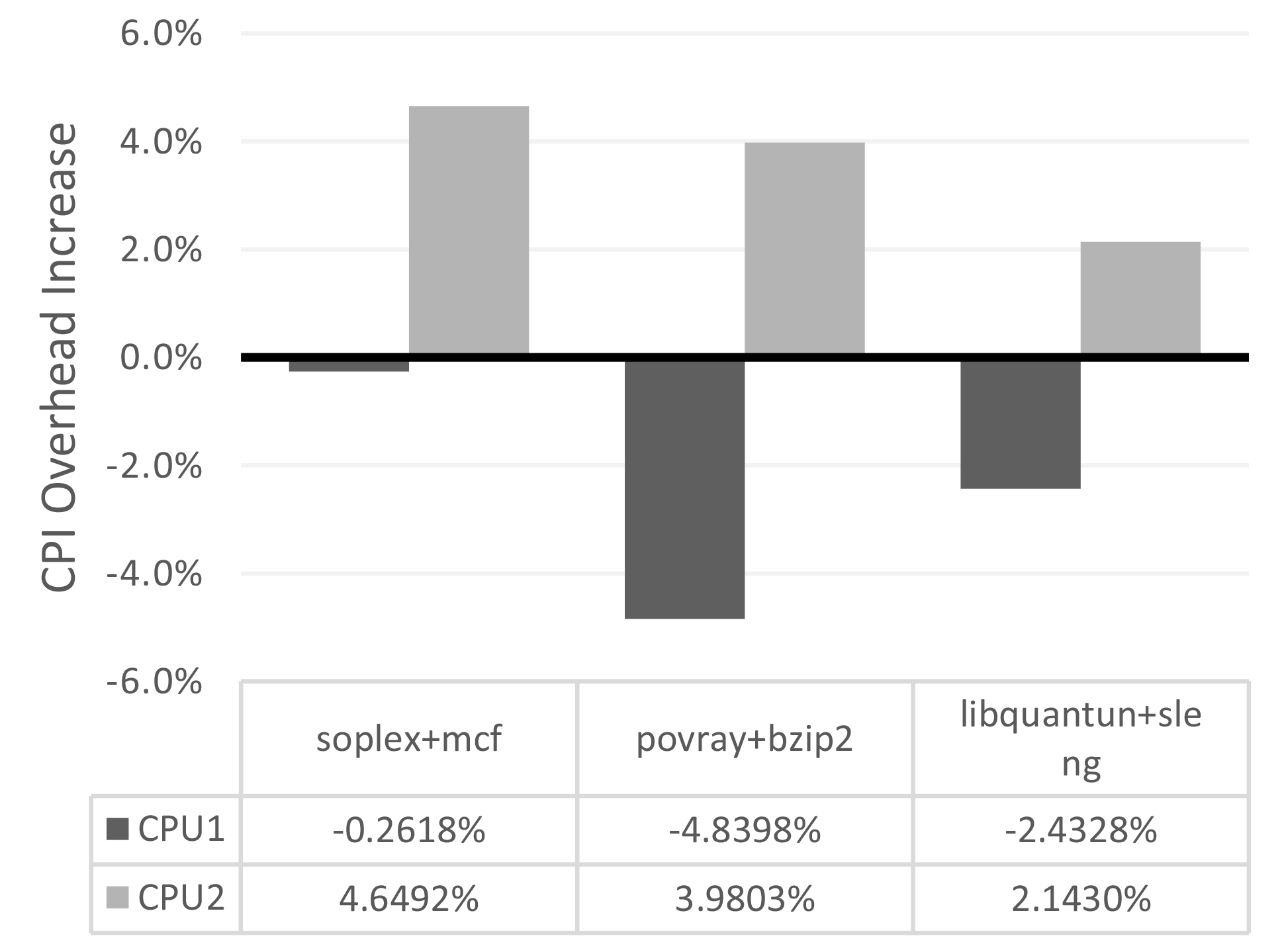

Supplement: S9 Fig — (TIF) [file pone.0201127.s009.tif]

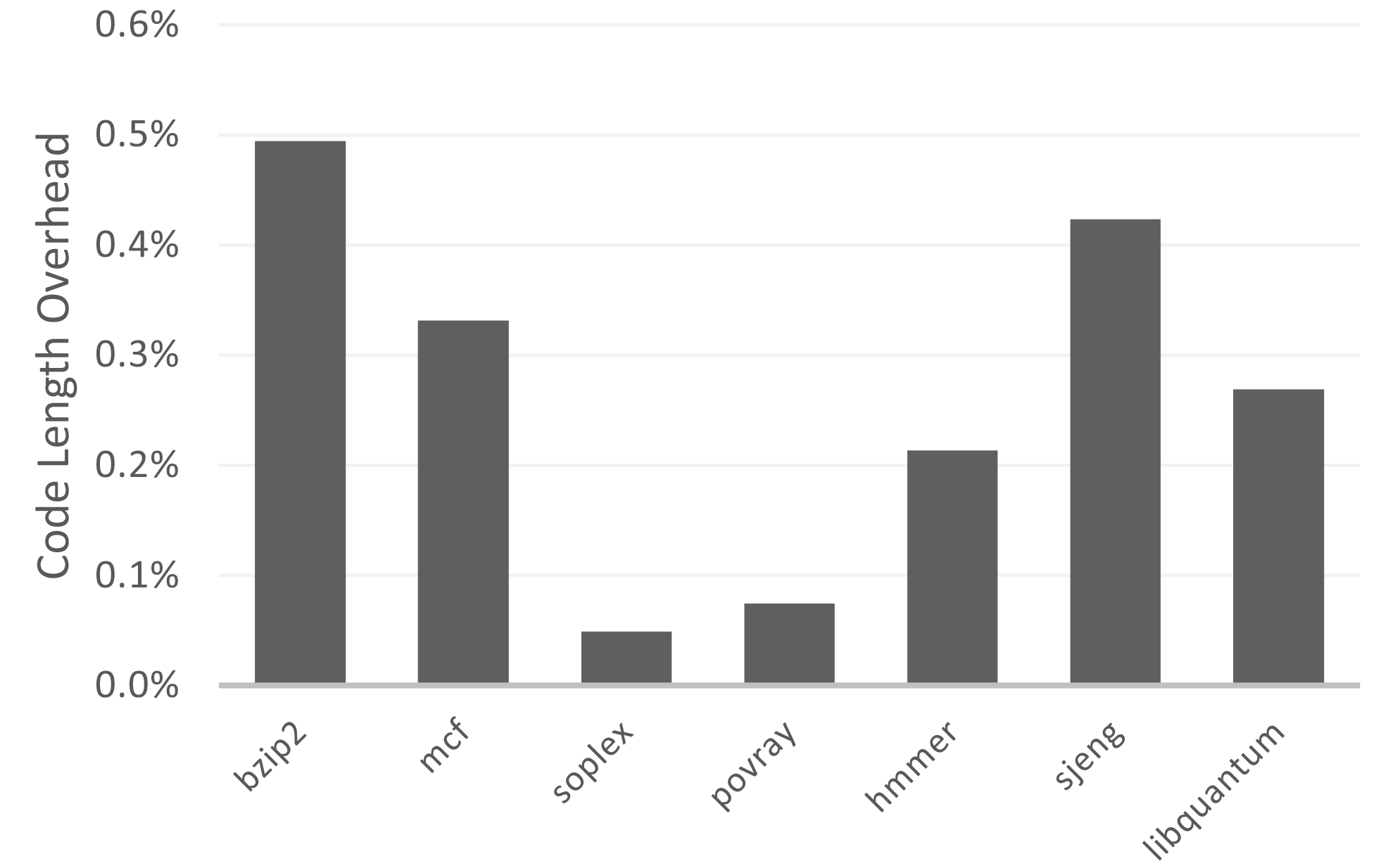

Supplement: S10 Fig — (TIF) [file pone.0201127.s010.tif]

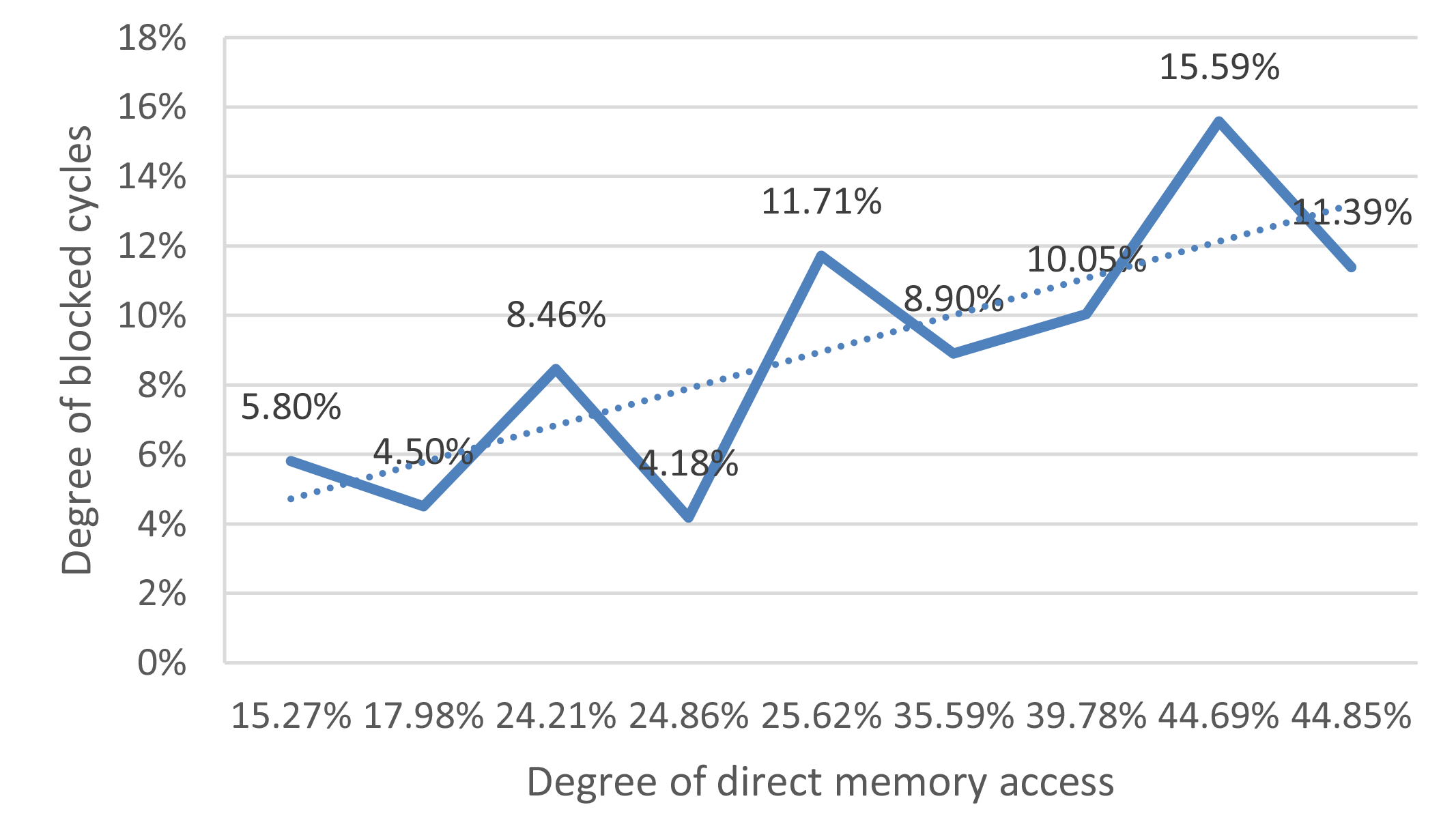

Supplement: S11 Fig — (TIF) [file pone.0201127.s011.tif]
